# A low cortisol response to acute stress is related to worse basal memory performance in older people

**DOI:** 10.3389/fnagi.2014.00157

**Published:** 2014-07-15

**Authors:** Mercedes Almela, Vanesa Hidalgo, Leander van der Meij, Matías M. Pulopulos, Carolina Villada, Alicia Salvador

**Affiliations:** ^1^Laboratory of Social Neuroscience, Department of Psychobiology, University of ValenciaValencia, Spain; ^2^Department of Social and Organizational Psychology, VU University AmsterdamAmsterdam, Netherlands

**Keywords:** cortisol, declarative memory, working memory, HPA-axis, elderly, older people, middle-age, acute psychosocial stress

## Abstract

Age-related memory decline has been associated with a faulty regulation of the hypothalamus-pituitary-adrenal axis (HPA-axis). The aim of this study was to investigate whether the magnitude of the stress-induced cortisol increase is related to memory performance when memory is measured in non-stressful conditions. To do so, declarative and working memory performance were measured in 31 men and 35 women between 55 and 77 years of age. On a different day, the magnitude of their cortisol response to acute psychosocial stress was measured. The relationship between the cortisol response and memory performance was U shaped: a low cortisol response to stress was related to poorer declarative and working memory performance, whereas those who did not increase their cortisol levels and those who had the largest cortisol increase had better declarative and working memory capabilities. Sex did not moderate these relationships. These results suggest that a low cortisol response to stress could reflect a defective HPA-axis response to stressors that is accompanied by poorer memory performance. Conversely, a high cortisol response seems to reflect a correct functioning of the HPA-axis and may protect against memory deficits in the later stages of human life.

## Introduction

There is great heterogeneity in the age-related cognitive decline among healthy people (Christensen et al., 1999). This means that some people maintain their memory relatively well as they age, while others experience a dramatic memory deterioration. It is crucial to unravel the causes for these individual differences in order to develop interventions that can improve quality of life among the elderly. One of the main body systems that has been associated with this heterogeneity is the activity and regulation of the hypothalamus-pituitary-adrenal axis (HPA-axis) and its end product, cortisol (e.g., Lupien et al., 2005). Many facets of the HPA-axis activity have been associated with memory capabilities in older people. For example, worse declarative memory performance has been related to increased basal cortisol levels (Lupien et al., 2009) and to an enhanced cortisol awakening response (Almela et al., 2012). The aim of this study was to further untangle the relationship between HPA-axis integrity and memory capacity in older people. In this study, we investigated whether the magnitude of cortisol reactivity to acute stress is related to basal memory performance, i.e., when memory is measured in non-stressful conditions.

Cortisol reactivity to stress may be related to basal memory performance because both the cortisol reactivity to stress and the memory performance are controlled by the same brain areas, namely the hippocampus and the prefrontal cortex. These two brain structures have a high density of receptors for cortisol, because they play a role in the feedback regulation of the HPA-axis activity (Herman et al., 2005). In fact, it has been shown that the magnitude of the cortisol response to stress is related to hippocampal volume (Pruessner et al., 2007) and to prefrontal cortex glucose metabolic rate (Liberzon et al., 2007; Kern et al., 2008). Therefore, the hippocampus and the prefrontal cortex control and shape the acute cortisol response to stress and, simultaneously, they are pivotal for cognitive processes such as declarative and working memory (Scoville and Milner, 1957; Galloway et al., 2008). It seems likely that a change in the functionality of either brain area will affect both the magnitude of the cortisol response to stress and memory performance.

Surprisingly, little is known about the relationship between the stress-induced cortisol response and memory performance under basal conditions in older people. In our opinion, it is crucial to know the nature of this relationship, since the magnitude of the cortisol response to acute stress could be an indicator of HPA-axis functionality. This indicator could therefore help us understand the memory decline observed in older people. What we do know is that aging is associated with a greater reactivity of the HPA-axis to pharmacological challenge, and that this has been explained as an age-related impairment in the HPA-axis negative feedback sensitivity (for reviews see: Seeman and Robbins, 1994 and Kudielka et al., 2009). Similarly, age has been associated with an increased reactivity to psychosocial stressors (Kudielka et al., 2004; Strahler et al., 2010; Almela et al., 2011b).

But, whether the magnitude of the stress-induced HPA-axis reactivity is related to “basal” memory capabilities is a question that remains unanswered, because research has focused mainly on the acute effects of stress on memory (e.g., Hidalgo et al., 2012; Pulopulos et al., 2013). Some preliminary evidence comes from two studies that found that high-cortisol responders to stress had poorer memory performance than non-responders both before and after the exposure to stress (Lupien et al., 1997), and that only in women, greater cortisol reactivity to psychosocial stress was related to poorer memory performance on a control day (Almela et al., 2011a). This suggests that responding to stress with a large cortisol increase is an indicator of faulty HPA-axis regulation, and that this relationship could be stronger in women than in men.

The goal of our study was to investigate among older people whether the magnitude of the cortisol response to stress is related to basal declarative and working memory performance as measured in a non-stressful condition. To do so, we selected a homogeneous healthy sample of men and women from 55 to 77 years of age. We included both men and women because we were interested in investigating the sex effects. This study involved two sessions. In the first session, a neuropsychological assessment of the participants was performed using two standardized tests for measuring declarative memory and another two for measuring working memory. Several days after the first session, the participants were exposed to the Trier Social Stress Test in a second session (TSST, Kirschbaum et al., 1993), which has consistently been shown to provoke endocrine, cardiovascular, immune and subjective stress responses (Allen et al., 2014). We expected that a higher cortisol response to stress would be related to poorer declarative and working memory performance.

## Materials and methods

### Participants

Sixty-five persons participated in this study (30 men and 35 women). Their age ranged from 55 to 77 years (men: *M* = 63.29, *SD* = 5.21; women: *M* = 63.54, *SD* = 3.66). Most of them were retired (91%) and had completed high school (85%). Men and women were not different in age or educational level (for both *p* > 0.5). We used the socio-economic status (SES) ladder to assess subjective SES (Adler et al., 2000: 1 = lowest to 10 = highest). Men scored slightly higher than women on the SES ladder (Men: *M* = 6.47, *SD* = 1.11; Women: *M* = 6, *SD* = 0.84, *p* = 0.064). Body mass index (BMI) was higher in men than in women (men: *M* = 27.80, *SD* = 3.96; women: *M* = 25.65, *SD* = 3.47, *p* = 0.023). All the women were postmenopausal (last menstruation at least one year before), and were not taking estrogen replacement therapy.

In total 166 persons volunteered for participation. We recruited these volunteers at university courses and seminars for retired people. They completed a questionnaire and were interviewed to assess if they complied with the inclusion criteria. Volunteers were not selected for participation if they met at least one of these criteria: alcohol or other drug abuse, smoking more than five cigarettes a day, visual or hearing problems, presence of a cardiovascular disease (e.g., angina), endocrine disorder (e.g., Cushing’s disease), neurological disease (e.g., epilepsy), cognitive disorder (e.g., dementia) or psychiatric disease (e.g., depression), the presence of a stressful life event during the last year (e.g., widowhood), having been operated with general anesthesia at least once in the past year and use of medication related to cognitive, emotional or endocrine function (e.g., antidepressants, benzodiazepines, glucocorticoids). To assess dementia we followed the criteria of DSM-IV and NINCDS-ADRDA criteria for Alzheimer’s disease. Sporadic use of painkillers and vitamins were allowed.

### Procedure

This study consisted of two different sessions, which participants had to attend individually. They were performed at two different locations at the Faculty of Psychology. The average time between sessions was 7 days (s.e. ± 0.42). The first session consisted of a neuropsychological assessment, and the second was a laboratory procedure employed to provoke an acute cortisol stress response (TSST). We decided that the neuropsychological assessment would always precede the stress session to avoid stress-related activation of the HPA-axis due to the anticipation and recall of the TSST. After participants had received verbal and written information about the study, they signed an informed consent form. The procedure of this study was approved by the Ethics Research Committee of the University of Valencia, and was conducted in accordance with the Declaration of Helsinki.

#### Neuropsychological assessments

The neuropsychological assessments were all conducted by the first author and lasted 01:30 h. Participants started this session either at 10:00 h (15 men and 18 women) or at 12:00 h (15 men and 17 women). Two saliva samples were provided to assess cortisol levels before and after the neuropsychological assessments (Pre-assessment and Post-assessment). Two tests were selected to assess declarative memory (Auditory Verbal Learning Test and Paragraph Recall) and another two tests were selected to assess working memory (Spatial Span and Spatial Working Memory). The selected tests have all shown good reliability and have been validated (Sahakian and Owen, 1992; Lezak et al., 2004).

##### Auditory verbal learning test

The WHO-UCLA Auditory Verbal Learning Test was administered (AVLT, Spanish version, Maj et al., 1994). This test contains a list of 15 neutral words (target list) that was read to the participants five times. After reading the list each time, the participants had to recall as many words as they could from the target list (learning trials). After this, an interference list was read (including 15 neutral words) and participants had to repeat as many words as they could from the interference list. Next, participants were asked to recall the target list (immediate recall after interference), and to recall it again after a delay of 30 min (delayed recall). Performance on this test was summarized using the following outcomes: (i) Total Learning: total words repeated in the five learning trials; (ii) Immediate Recall: total words of the target list recalled after the interference; and (iii) Delayed Recall: total words of the target list recalled after the 30 min delay.

##### Paragraph recall

The Spanish version of the Logical Memory test was administered (Wechsler Memory Scale III; Pereña et al., 2004). The experimenter read out loud two brief narratives and participants had to recall as many contents or “ideas” as possible from these narratives, immediately after having heard them (immediate trials), and after a 25 min delay (delayed trials). Performance on this test was summarized using the following outcomes: (i) Immediate recall: total ideas recalled at the immediate trials; and (ii) Delayed recall: total ideas recalled at the delayed trials.

##### Spatial span

This test is a computerized version of the Corsi Block Tapping Task that is applied using a tactile computer screen. It is a subtest of the Cambridge Neuropsychological Test Automated Battery (CANTAB, Cambridge Cognition, Cambridge, United Kingdom, 2006)^1^ and measures short term memory span. Participants had to remember the correct sequence in which a variable amount of squares (from 2 until 9) had changed their color on the screen. In the first part, they had to reproduce the sequence in the same order (forward), and in the second part in reverse order (backward). If the participants failed to reproduce the correct sequence twice, the test stopped. Performance on this test was summarized using the following outcomes: (i) Spatial Length Forward: highest number of squares which color change sequence could be reproduced in the same order; and (ii) Spatial Length Backward: highest number of squares which color change sequence could be reproduced in reverse order.

##### Spatial working memory

This test is a subtest of the CANTAB that measures spatial working memory (Owen et al., 1995). A tactile computer screen was filled with several boxes. Participants were told that they had to find a rectangle that was hidden beneath one of these boxes. Once they found the rectangle, they were told that they had to find another rectangle, but that this time the rectangle could not be beneath a box where a rectangle had already been found. The outcome of this test was the total errors committed, which was the sum of errors committed by touching a box that had been found empty in the same trial, and errors committed by touching a box where a rectangle had already been found in a previous trial.

#### Cortisol stress response: trier social stress test

The TSST was used to provoke stress. The session took 01:50 h, and was always carried out in the afternoon in three different shifts starting at either 16:00 h, 17:15 h or 18:30 h. The distribution of men and women was similar in all three shifts (*p* > 0.3). Several instructions were given to the participants before coming to this session: from the day before this session they had to maintain their usual habits and sleep pattern, not engage in activities which involved heavy physical activity, and they could not drink alcohol. Additionally, up to 2 h before this session they were instructed only to drink water, and not smoke, eat or take any stimulants (e.g., cola, tea, chocolate). When participants arrived to the lab, the experimenter asked them if they had followed these instructions.

Next, participants were accompanied to Room A where they remained seated during 15 min (habituation phase). The first saliva sample was provided 5 min before this phase ended (−20 min). Then, they were accompanied to Room B. In this room there were a man and a woman sitting in front of them (committee), and a microphone and a video camera clearly visible. Participants were instructed that they had to perform a speech in front of this committee (introduction phase). Next, they had 10 min to prepare for the speech, which they did in Room A (preparation phase). The second saliva sample was provided just before the onset of the speech (0 min). Participants were accompanied to Room B were they performed the free speech during 5 min, and an arithmetic task during 5 min. Both tasks were filmed. Afterwards, participants were accompanied to Room C where they remained 60 min (recovery phase), during which they answered some questionnaires and rested. Four saliva samples were provided during the recovery time: +25 min, +40 min, +55 min and +70 min. After the last saliva sample, the protocol ended and the participants were debriefed.

### Heart rate

Heart rate (HR) was measured with a Polar HR monitor during the entire course of the stress session (Polar, model S810i, Electro Ltd., Kempele, Finland). To this end, participants wore a chest belt for HR detection and a watch to store HR data. The accuracy of the Polar HR monitor was 1 ms, and it has been shown that measurements from these types of monitors have good validity (e.g., Radespiel-Tröger et al., 2003). Artifacts were removed and the recorded periods in which participants changed their orthostatic load (sitting/standing up) or when they were walking were also removed. For the final analyses we used the HR mean for each phase of the experiment.

### Saliva sampling and biochemical analyses

To asses cortisol levels participants provided six saliva samples by passively drooling 3 ml of saliva in plastic vials (+/− 5 min to complete). The saliva samples were frozen at −20°C until analyses. The Laboratory of Social Neuroscience in the Central Research Unit of the Faculty of Medicine at the University of Valencia assayed the samples for cortisol with a competitive solid phase radioimmunoassay (tube coated), using the commercial kit Spectria Cortisol RIA kit (cat. no. 06119) from Orion Diagnostica (Espoo, Finland). Assay sensitivity was 0.8 nmol/l. For each participant, all the samples were analyzed in the same trial. The within and inter assay variation coefficients were all below 8%.

### Statistics

Student’s *t*-test and Chi-square analyses were used to investigate sex differences in the demographic variables. Significant deviations from normality were detected in cortisol and HR values, and thus values were square root transformed. Two participants were excluded from the analyses involving HR (two women) and cortisol (a man and a woman) because their HR and cortisol concentrations differed by more than 3 S.D. from sample mean.

To analyze the cortisol response to the neuropsychological assessment we performed an ANOVA for repeated measures. To analyze the cortisol and HR responses to the stress induction, we performed ANOVAs for repeated measures. As men and women differed slightly in their subjective SES and BMI, we explored whether these variables were related to their cortisol and HR responses to the TSST. Results showed that SES and BMI were not correlated to the cortisol levels during the neuropsychological assessment nor to the cortisol or HR responses to stress (for all *p* ≥ 0.107), therefore these variables were not included as covariates. If the requirement of sphericity was not met in the ANOVA for repeated measures, we used the Greenhouse-Geiser correction. *P*-values were corrected with the Bonferroni correction when testing *post-hoc* planned comparisons.

To analyze whether the memory performance was different depending on the cortisol response to stress, we performed regression analyses using cortisol AUCi (change in total cortisol levels from baseline, see Pruessner et al., 2003 for the specific formula) as a measure of cortisol reactivity.^2^ We included sex as moderator, according to the procedure of Aiken and West (1991), to investigate if sex moderated the relationship between cortisol reactivity and memory performance. Scatterplots were inspected to investigate whether the relationships were linear or curvilinear. All *p*-values reported are two-tailed, and the level of significance was marked at <0.05. When values are not specified they are mean ± standard error of mean (SEM). Analyses were performed with SPSS 19.0.

## Results

### Heart rate response to the TSST

**Figure 1:**
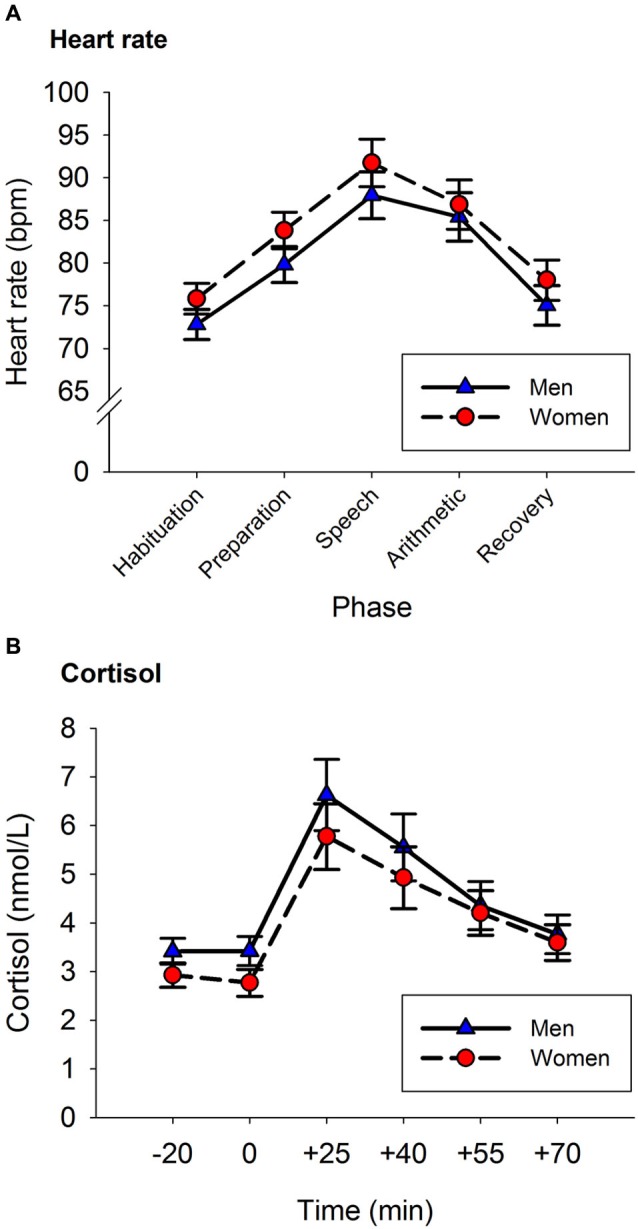
**Heart Rate (A) and Cortisol (B) responses to the TSST.** Panel **A**: Heart rate increased from baseline until reaching peak levels during the speech phase. Afterwards, HR decreased reaching baseline levels in the recovery phase. Panel **B**: Cortisol levels increased from baseline until reaching peak levels at the +25 sample. Afterwards, cortisol levels decreased reaching baseline levels in the last saliva sample. Error bars represent SEM.

A repeated-measures ANOVA was used to analyze the HR response to the TSST. Phase was entered as a within-subject factor (Habituation, Preparation, Speech, Arithmetic and Recovery), and Sex was entered as a between-subject factor. The results showed a main effect of Phase, *F*_(2.80, 162.85)_ = 67.442, *p* < 0.001 (see Figure 1A). The factor Sex was not significant, nor was there an interaction between Sex and Phase, both *p* > 0.3. The participants’ HR increased from baseline to the speech phase ( *p* < 0.001), and steadily decreased from the speech phase to baseline levels in the recovery phase (recovery phase vs. baseline, *p* > 0.8).

### Acute cortisol response to the neuropsychological assessment and to the TSST

A repeated-measures ANOVA was used to analyze the impact of the neuropsychological assessment on the participants’ cortisol levels. Time (pre- vs. post-assessment) was included as within-subject factor, and Sex as a between-subject factor. Following the circadian rhythm of cortisol, participants’ cortisol levels were lower at the end of the assessment than at the start of the assessment (pre-assessment: 5.14 (0.3) nmol/L, post-assessment: 3.99 (0.2) nmol/L, *F*_(1,61)_ = 10.973, *p* = 0.002). Sex did not have any effect on the release of cortisol (for all *p* > 0.2).

A repeated-measures ANOVA was used to analyze the acute cortisol response to the TSST. Time was entered as a within subject factor (−20, 0, +25, +40, +55, +70) and Sex as a between-subject factor. There was a main effect of Time, *F*_(1.78, 108.42)_ = 32.003, *p* < 0.001. The factor Sex was not significant, nor was there an interaction between Sex and Time (for both *p* > 0.2).

Figure 1B shows the amount of cortisol released by men and women before and after the stress induction. Cortisol concentrations were not different in the two samples provided before the stress task (*p* > 0.9). The peak of cortisol concentration was reached 25 min after the onset of the stress task (+25 vs. −20: *p* < 0.001). Afterwards, cortisol concentrations decreased until reaching similar levels to those of the baseline sample in the last saliva sample (+70 vs. −20: *p* > 0.9).

### Relationship between the stress-induced cortisol response and memory performance

To test whether there was a relationship between the stress-induced cortisol response and basal memory performance, we performed hierarchical regression analyses (Aiken and West, 1991). The inspection of the scatterplots suggested that some relationships could be curvilinear; therefore, a curvilinear term was added in the analyses. We performed separate analyses for each memory outcome being predicted by AUCi. In step 1, we added the following control variables: age, BMI, SES, mean of the cortisol levels during the neuropsychological assessment, basal cortisol levels at the TSST session, and sex (0 = women, 1 = men). In step 2, we added AUCi to investigate a linear relationship between the memory outcome and cortisol secretion. In step 3, we added the square of AUCi to investigate a curvilinear relationship between memory outcome and cortisol secretion. In step 4, we added the interaction term Sex*AUCi, and in step 5 we added the interaction term Sex*AUCi^2^ to investigate whether the relationship between the cortisol response and memory was moderated by the sex of the participants. All predictors were standardized prior to entry into the regression analyses to facilitate the interpretation of first-order terms and reduce multicollinearity. When a significant curvilinear relationship is found, a positive *β* represents a concave upward relationship (U-shaped form) and a negative *β* represents a concave downward relationship (inverted U-shaped form). Results of these regression analyses are summarized in Table 1.

**Table 1:** **Short summary of the relationships found between basal memory test outcomes and cortisol AUCi in response to acute psychosocial stress**.

**Memory Test**	**Outcome**	**AUCi**
Auditory Verbal	Total Learning	–
Learning Test	Immediate Recall	–
	Delayed Recall	–
Paragraph Recall	Immediate Recall	♂♀: U-shaped
	Delayed Recall	♂♀: U-shaped
Spatial Span	Forward	♀: U-shaped
	Backward	♂: U-shaped
Spatial Working Memory	Total Errors	♂♀: Inverted U-shaped

#### Auditory verbal learning test

There were no statistically significant linear or curvilinear relationships between cortisol AUCi and any memory outcome of the AVLT (for all *p* > 0.080).

#### Paragraph recall

**Figure 2:**
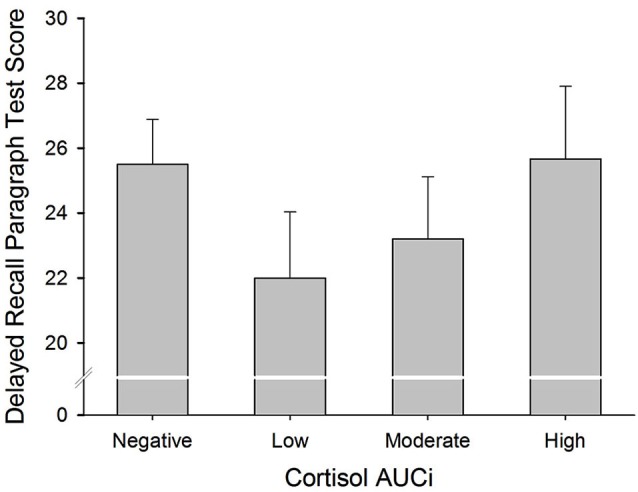
**Delayed Paragraph Recall performance and cortisol reactivity to stress.** Participants were divided into four groups: (i) Negative AUCi; (ii) Low: first tertile positive AUCi; (iii) Moderate: second tertile positive AUCi; and (iv) High: third tertile positive AUCi.

There was a curvilinear relationship (U-shaped) between cortisol AUCi and performance on both the immediate and delayed recall trials of the paragraph recall test (Immediate Recall: Adj *R^2^* = 0.058, Δ*R^2^* = 0.078, *β* = 0.307, *F*_(1,54)_ = 5.101, *p* = 0.028; Delayed Recall: Adj *R^2^* = 0.069, Δ*R^2^* = 0.077, *β* = 0.307, *F*_(1,54)_ = 5.152, *p* = 0.027). These results indicate that those participants who did not show a cortisol response (a negative AUCi) or showed a large cortisol response to stress performed better on this test than those who had a low cortisol increase after stress induction (see Figure 2). Sex did not moderate the relationship between cortisol release and performance on this test (for all *p* > 0.1).

#### Spatial span and spatial working memory

**Figure 3:**
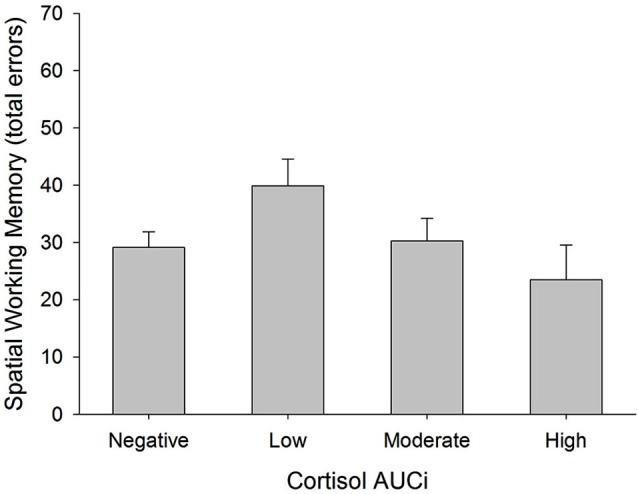
**Total errors in Spatial Working Memory test and cortisol reactivity to stress.** Participants were divided into four groups: (i) Negative AUCi; (ii) Low first tertile positive AUCi; (iii) Moderate: second tertile positive AUCi; and (iv) High: third tertile positive AUCi.

There was a curvilinear relationship between cortisol AUCi and performance on the spatial working memory test (total errors: inverted U-shaped form, Adj *R^2^* = 0.186, Δ*R^2^* = 0.067, *β* = −0.286, *F*_(1,54)_ = 5.125, *p* = 0.028). Therefore, the better performance on this test was achieved by those participants who showed either no cortisol response (negative AUCi) or a large cortisol response to stress. Those participants with a small cortisol increase achieved a worse performance on this test (see Figure 3). This same curvilinear relationship was also found for the forward spatial length only in women (Adj *R^2^* = 0.177, Δ*R^2^* = 0.055, *F*_(1,52)_ = 4.118, *p* = 0.048, men: *β* = 0.085, *p* > 0.5, women: *β* = 0.698, *p* = 0.013), and for the backward spatial length only in men (Adj *R^2^* = 0.092, Δ*R^2^* = 0.087, *F*_(1,52)_ = 5.965, *p* = 0.018, men: *β* = 0.379, *p* = 0.016, women: *β* = −0.396, *p* > 0.1).

## Discussion

This study investigated whether the magnitude of the HPA-axis response to acute stress is related to basal memory capabilities among men and women between 55 and 77 years of age. We did so by measuring the participants’ declarative and working memory performance in a non-stressful condition and, on a different day, we measured their cortisol response to psychosocial stress. Results showed that a low cortisol response to stress was related to poorer declarative and working memory performance, whereas no increase in cortisol levels and a large cortisol increase were related to better declarative and working memory performance.

Our results were not in line with our hypothesis, as we expected a poorer memory performance among those who reacted to the TSST with larger cortisol increases. Nevertheless, our hypothesis was based on other studies that did find that an enhanced basal cortisol secretion was linked to worse memory performance among older people (e.g., Lupien et al., 2005). Furthermore, other studies have found that non-cortisol responders had better memory performance than cortisol responders, both before and after a stress induction procedure (Lupien et al., 1997), and that only among women, a higher cortisol response to stress was related to worse performance on the first trials of the AVLT in a control session (Almela et al., 2011a). An explanation for these divergent results could be that these last two studies were originally designed to investigate acute effects of stress on memory performance, and thus did not include enough participants to test for curvilinear relationships.

But, why and how would a low cortisol response to acute stress be related to worse basal memory performance? In our opinion, this can be best explained by taking into account the adaptive function of cortisol when facing a threat, regardless of whether the threat is to our physical or our social well-being (Dickerson and Kemeny, 2004). In fact, the release of cortisol initiates a series of physiological changes that have the purpose of improving our performance (e.g., deviation of energy to our muscles, the enhancement of our cardiovascular tone, the suppression of functions that are not essential at that moment) (Sapolsky et al., 2000). Therefore, it seems plausible that once a stimulus has been evaluated as threatening, having a low cortisol response can constitute a maladaptive response because it can reduce our chances of success. In support of this, a reduced HPA-axis response to stress has been observed in several stress-related pathologies such as atopic dermatitis, asthma (Buske-Kirschbaum et al., 2002, 2003) and, interestingly, also in depression, which in turn is accompanied by declarative and working memory deficits and a decrease in hippocampal and prefrontal cortex volumes (Burke et al., 2005; Savitz and Drevets, 2009).

Hence, the results of our study suggest that a low cortisol response to stress is evidence of not optimally functioning HPA-axis which is accompanied by poorer basal memory performance among healthy older people. The literature on this matter is very scarce, but a study performed in young individuals supports this notion by showing that a higher cortisol response to stress was associated with larger hippocampal activation in an encoding task performed before stress exposure (Khalili-Mahani et al., 2010). This increase in hippocampal activation suggests better memory performance as many other studies have shown that higher hippocampal activation during encoding is followed by better recovery of the material previously learned (e.g., Zeineh et al., 2003; Carr et al., 2010).

A possible mechanism through which a low cortisol response to stress can be a risk factor for memory problems can be related to the long-term effects of cortisol. Thus, cortisol shapes and restrains other stress-related physiological processes that can have detrimental effects on our organism if they continue to be activated once the stressor has been overcome, such as immune responses and the release of the corticotrophin releasing hormone (CRH; Sapolsky et al., 2000; Raison and Miller, 2003). In fact, unrestrained inflammation and CRH release secondary to insufficient cortisol-mediated feedback inhibition influence cell survival in the central nervous system and contribute to neuronal degeneration (Allan and Rothwell, 2001; Nadeau and Rivest, 2003). This notion is supported by the finding that a low cortisol response to the TSST among healthy older people is associated with the occurrence of more stressful life events during childhood and adolescence and the presence of the G allele on the serotonin receptor gene 1A (HTR1A G) (Armbruster et al., 2011). In fact, both the HTR1A G allele and more stressful life events during childhood have been associated with stress-related pathologies (i.e., depression, PTSD, anxiety, Heim and Nemeroff, 2001; Savitz et al., 2009), lower memory function and reduced volumes of hippocampus and prefrontal cortex (Dannlowski et al., 2012; Yen et al., 2013).

Apart from those who had the largest cortisol response to the TSST, those who did not increase their cortisol levels in response to the stressful situation also had better memory performance. It is possible that the TSST was not stressful enough to trigger a cortisol response among these participants. Therefore, a protective trait against memory deficits could be having a higher threshold for triggering stress-related HPA-axis activation. However, it is also possible that, giving the cognitive nature of the TSST, those participants with higher cognitive capabilities were less stressed by the TSST.

We found some sex differences in the strength of the relationship between the stress-induced cortisol response and basal memory performance (see Table 1). Moreover, the stress-induced cortisol response was not related to the outcomes of the AVLT. In our opinion, these sex differences were not robust, since the same curvilinear relationships between the outcomes of the paragraph recall test and the spatial working memory test were found in both sexes. It is possible that some memory tests would be more sensitive than others to show differences in performance related to cortisol reactivity to stress. Because of that, it is advisable in future studies to use a wide range of memory tests.

In our study we did not find sex differences in the cortisol response to the stress induction. This finding is not in agreement with previous research that showed that men reacted to the TSST with a larger cortisol increase than women (Kudielka et al., 2004; Strahler et al., 2010; Almela et al., 2011b). Additionally, we consider that the overall cortisol response to the TSST was moderate because the mean peak of cortisol was 6.5 nmol/l, whereas in the later studies cortisol concentrations have reached 10 nmol/l or more. When we compare the protocol of the current study with our previous protocol (Almela et al., 2011b), there are several differences that can explain these discrepancies. First, the age of the TSST committee was different in the two studies. The age of the audience in the current study was between 25 and 30 years old, whereas in Almela et al. (2011b) the age of the audience was similar to that of the participants. We consider that performing the speech and arithmetic task in front of a younger audience could reduce its stressfulness. Second, the exposure to the TSST was always carried out several days after the neuropsychological assessment. It is possible that having participated in a neutral session before the stress session had an effect on the expectations of the participants, as well as reducing the novelty effect. Nonetheless, we tried to differentiate between the two sessions by performing them in different laboratories with different experimenters. Thus, it could be that in the current study, especially men were more sensitive to these small variations in the protocol and consequently responded with a lower cortisol increase to the stress induction. This resulted then in a similar cortisol response to stress in men and women. Future studies, should take these protocol differences into account, especially when strong cortisol responses to stress are sought.

Some limitations have to be considered in interpreting the results of the current study. The sample consisted of people who were very healthy for their age, because we wanted to avoid as many confounds as possible by selecting the participants using very restrictive health- and medication-related criteria. However, this resulted in a moderate sample size and this selection procedure can be problematic for the generalizability of the results. Future research could use other populations composed of people with specific age-related health problems. Furthermore, the results of our study emphasize the importance of including enough participants in future studies to have enough power to detect curvilinear and linear relationships. In addition, we have to mention the correlational nature of this study, which does not allow for endorsing causal relationships. Therefore, it is important that our results are replicated using, for example, longitudinal designs.

Taken together, our results show that among healthy older individuals both declarative and working memory capabilities are higher when they do not respond with a cortisol increase to acute stress or they respond with a large cortisol increase. A low cortisol response to stress was related to worse memory performance. These findings suggest that a large cortisol response to acute stress reflects a well-functioning HPA axis, and may protect against memory deficits in the later stages of human life.

## Conflict of interest statement

The authors declare that the research was conducted in the absence of any commercial or financial relationships that could be construed as a potential conflict of interest.

## Footnotes

^1^www.cantab.com

^2^We also performed regression analyses using Delta Change (maximum cortisol levels reached after the exposure to stress minus baseline levels) instead of AUCi as a measure of cortisol reactivity. The statistical conclusions from these analyses were not different from the analyses using AUCi. Therefore, we decided to report only the results using AUCi.
